# Oxidative stress constrains evolution of bacteriophage host-range diversity

**DOI:** 10.1093/ismejo/wrag090

**Published:** 2026-04-14

**Authors:** Coline Meynard-Doumenc, Quentin Lamy-Besnier, Loïc Brot, Marie Messika, Auguste Wolfromm, Jean-Pierre Grill, Luisa De Sordi

**Affiliations:** INSERM, Centre de Recherche Saint-Antoine, Sorbonne Université, 75012 Paris, France; INSERM, Centre de Recherche Saint-Antoine, Sorbonne Université, 75012 Paris, France; INRAE, MICALIS Unit, Université Paris-Saclay, 78352 Jouy-en-Josas, France; INSERM, Centre de Recherche Saint-Antoine, Sorbonne Université, 75012 Paris, France; INSERM, Centre de Recherche Saint-Antoine, Sorbonne Université, 75012 Paris, France; INSERM, Centre de Recherche Saint-Antoine, Sorbonne Université, 75012 Paris, France; INSERM, Centre de Recherche Saint-Antoine, Sorbonne Université, 75012 Paris, France; INSERM, Centre de Recherche Saint-Antoine, Sorbonne Université, 75012 Paris, France

**Keywords:** phage, reactive oxygen species, antagonistic coevolution, *Escherichia coli*

## Abstract

Reactive oxygen species (ROS) are essential for cellular signalling and redox homeostasis, but their accumulation causes cellular oxidative stress. In inflammatory bowel disease, oxidative stress is linked to chronic inflammation and alterations in the gut microbiota. We hypothesised that these alterations may result from the impact of ROS on the interactions between bacteria and their viruses, bacteriophages. We followed the evolution of three *Escherichia coli* strains and a virulent bacteriophage in a chemostat under continuous growth and studied the impact of oxidative stress on this community. We show that both the bacteriophage and its three hosts persisted in the system over 10 days, but the relative abundance of bacteriophages was decreased in the presence of ROS. Oxidative stress also limited bacteriophage population diversity by favouring the selection of specialist bacteriophages with a narrower host range. Concomitantly, ROS accelerated the evolution of bacterial resistance to bacteriophages and drove the fixation of genomic mutations in genes related to cell surface structures or located in mobile genetic elements. These results highlight that oxidative stress impacts the evolutionary dynamics between bacteria and bacteriophages with consequences for microbiota diversity and potential implications in the context of intestinal inflammation.

## Introduction

Reactive oxygen species (ROS) play a central role in the physiological regulation of intracellular signalling pathways and inflammatory response [1, 2]. However, when antioxidant mechanisms fail to neutralize ROS, their accumulation in the extracellular and intracellular environment leads to oxidative stress. Chronic oxidative stress can result in cellular and organ damage and is associated with chronic inflammation, as seen with excessive ROS production from overactivated phagocytic cells in inflammatory bowel disease (IBD) [3].

In IBD, oxidative stress impacts the physiology of both the host and the gut microbiota. Strictly anaerobic intestinal bacteria are particularly sensitive to oxidative stress, which might partly explain why the microbiota of IBD patients is characterized by a decreased bacterial diversity and an increase in facultative anaerobes, like *Enterobacteriaceae* [4–7]. Oxygen-tolerant bacteria are equipped to deal with endogenous and exogenous ROS [8, 9], through inducible detoxifying enzymes like catalases and superoxide dismutase under the control of regulatory systems like the OxyR regulon in *Escherichia coli*. Nevertheless, high ROS concentrations can negatively impact bacterial growth, primarily via DNA and protein damage [10].

Bacteria also face selective pressure from their viral parasites, bacteriophages (or phages), and carry different defence mechanisms to counteract this pressure [11, 12]. The evolutionary arms race between phages and bacteria was intensively studied *in vitro* and *in vivo,* revealing complex dynamics that drive the maintenance or the expansion of their phenotypic and genomic diversity [13–15].

Intestinal phages show temporal stability similar to that of bacteria, suggesting a coexistence of these populations [16, 17]. The nature of this coexistence is thought to be individual-specific and sustained by different dynamics like antagonistic coevolution or the spatial segregation of predator and prey populations [18]. The bacterial metabolic or stress state are also determinants of phage susceptibility in the gut [19] but the effect of ROS on phage-bacteria interactions has been largely overlooked.

Studies on *Campylobacter jejuni* suggest that oxidative stress response is associated with decreased phage infectivity [20] and phage infection may, in turn, induce expression of oxidative stress response genes in multiple bacteria [21]. In lysogens, ROS can induce prophage excision resulting in Shiga-toxins production in *E. coli* or inhibition of the oxidative stress response in *Salmonella* [22]. Despite these findings in specific bacterial-phage pairs, the role of ROS in complex microbial communities, especially within the context of microbiota-associated phages, remains understudied [14, 23].

In a previous study using a simplified community model consisting of a single virulent phage and three *E. coli* strains, we demonstrated that a community context facilitates the diversification of phage host range. Indeed, the availability of divergent infection pathways via different bacterial hosts allowed phage evolution and adaptation to multiple bacterial hosts *in vitro* and in the gut of mice [24]. In this study, we hypothesise that oxidative stress can directly or indirectly influence phage infectivity and host range, thus altering microbiota diversity. We used the same synthetic community grown in continuous culture in chemostats in presence or absence of ROS and showed that oxidative stress restrains the expansion of phage diversity and host-range while increasing bacterial resistance to phage infection.

## Materials and methods

### Bacterial strains and phages

*Escherichia coli* strains MG1655 (GFP, AmpR, StrepR), LF82 (ΔampC, dsRed, KanR), and MEc1 [24] were routinely cultured in lysogenic broth (LB, Sigma L3022) or on LB agar at 37°C. The bacteriophage LF82_P10 (termed P10 in this study) was isolated from *E. coli* LF82 strain [25]. The phage suspension was amplified on strain LF82 at a multiplicity of infection of 0.1:1, diluted in TN buffer (Tris 10 mM, NaCl 100 mM), and stored at 4°C.

### Chemostat conditions

Two millilitres of culture (OD_600nm _= 0.8) of each bacterial strain were inoculated into the 200 ml LB medium in Lambda Minifor fermenter. The pH was adjusted to 7, the temperature was set to 37°C and the medium was shaken continuously by up-and-down agitation with ‘fish-tail’ stirring discs (Lambda). A continuous culture was set up with a constant flow of 70 ml/h of LB medium added and removed from the fermenter using two peristaltic pumps. After 48 h, 200 μl of phage P10 suspension at 10^9^ pfu/ml was added, giving a final concentration of 10^6^ pfu/ml. For experiments under oxidative stress, a continuous flow of 440 μl/h of 3% hydrogen peroxide (Sigma H1009) was added to obtain a constant concentration of 500 μM in the fermenter medium. The experiments were carried out over 11 days and 1.5 ml samples were removed daily and stored at 4°C until used.

### Bacteria and phages enumeration

To separate the phages from the bacteria, each chemostat sample was centrifuged for 5 min at 2500 g. The supernatant, containing the phages, and the pellet, containing the bacteria, were separated. The phage fraction was treated with chloroform and the pellet was resuspended.

Bacteria were serially diluted and plated on selective LB agar. Kanamycin was added at a concentration of 100 μg/ml for the LF82 strain selection, 100 μg/ml of ampicillin for the MG1655 strain, and 25 μg/ml of chloramphenicol for the MEc1 strain. As the MEc1 strain is multi-resistant, it was selected on ampicillin with strain MG1655. Colonies of this strain were then isolated by detection of GFP protein expression using a Dark Reader transilluminator (Clare Chemical Research). Phages were enumerated by plaque assay, i.e. phages were spotted on a bacterial lawn of each strain to observe lysis, resulting in three subpopulations of phages. Each plate was incubated at 37°C overnight and then bacterial colonies (cfu) and lysis plaques (pfu) were enumerated. Adsorption of phage P10 to bacterial clones was measured by adding 2 × 10^6^ pfu to exponentially growing cultures (OD_600nm_ = 0.3) and redrawing aliquots every 2 min during 10 min to titer the unabsorbed phage [26].

To evaluate the impact of hydrogen peroxide on phage titration or bacterial growth, 500 μM H_2_O_2_ (Sigma) was added to exponentially growing bacterial culture or phage lysate at a concentration of 10^9^ pfu/ml. Bacteria were inoculated in 96 wells plate, incubated at 37°C and optical density measurements were automatically taken every 15 min during 10 h using an Infinite M200 plate reader (Tecan). The P10 phage was incubated at 37°C with hydrogen peroxide and titrated by plaque assay after 6 h. All experiments were performed in three biological replicates. Bacterial growth rates (μ) were calculated as the slope of the natural logarithm (Ln) of the OD_600nm_ values matching the exponential phase. Comparison of growth rates in the presence or absence of H_2_O_2_ was performed for each strain using a Welch’s t-test.

To test for rapid selection of bacterial variants after exposure to oxidative stress we cultured strains LF82, MG1655 for 16 h in the presence of 500 μM H_2_O_2,_ and genomic DNA was extracted from six to eight clones per strain, re-isolated on LB solid media. Genes *ompR, ompC*, and *waaO* were amplified (T100 Thermo-cycler—BioRad) and Sanger sequenced (Eurofins) with forward and reverse primers to cover the full length of the gene. Sequences were aligned with MUSCLE v5 [27] to the reference genes of each strain.

To test for bacterial susceptibility to increasing doses of H₂O₂, strains were grown in fresh medium to OD_600nm_ = 0.5. H₂O₂ was diluted in sterile water to 0, 0.5, 0.75, 1, and 2 mM immediately before adding 100 μl of culture. Bacteria were incubated 15 min at room temperature before enumeration on LB agar plates.

### Time-shift experiments

Eighty clones of each bacterial strain and of each subpopulation of phages infecting the three bacterial strains were isolated from CFU or PFU from samples of Days 4, 7, and 10 and resuspended in LB medium and TN buffer respectively. Bacterial clones were conserved in 15% glycerol solution at −80°C and phage clones in TN buffer at 4°C. The phage clones were firstly tested on ancestral strains to determine the phage infection capacities by spotting a non-diluted phage suspension onto LB agar plates covered with a bacterial lawn. Then, eight clones of each bacterial strain and 24 clones of phage P10 with different infectivity profiles were selected from samples at Days 4, 7, and 10 and a time-shift experiment was realised to estimate the coevolution within chemostats. Coevolution was estimated over three time-points of bacteria and phage, Days 4, 7, and 10. Changes in phage infectivity/bacteria resistance were tested through spot assay, as described above. Each phage clone was spotted onto lawns of individual past, present, and future bacterial clones. Plates were incubated overnight at 37°C and phage clones were scored as infectious (1) or not (0) by observing the appearance of lysis.

### Time-shift statistical analysis

The time-shift data were analysed through the phage infectivity towards each bacterial clone, which is the percentage of phages that were able to infect the clone. Changes in phage susceptibility were analysed using an approach adapted from a published method [28]. Data were modelled using a generalized linear mixed effects model, using a binomial error structure. Two models were built: the first one did not include the different *E. coli* strains as a parameter in the model, and the second did. Both models were treated similarly. The random effects included the fermenter from which the clone was isolated, as well as the clone itself, which was added in order to control the model overdispersion [29]. The models were simplified by removing non-significant interactions using likelihood ratio tests. The post-hoc tests were then performed on the most parsimonious models. The analysis was performed with R version 4.3.2 [30], using the package lme4 [31] for the modelling, and the package emmeans [32] for Tukey’s post-hoc tests. The threshold for statistical significance was set at *P* < .05 for all analyses. The code corresponding to this analysis can be accessed at https://doi.org/10.5281/zenodo.17640478.

### DNA extraction

Phages were amplified on their isolation strain to a sufficient titer (10^7^–10^9^ pfu/ml) before being treated with chloroform (10% v/v) and lysates were filtered through a 0.45 μm filter. The lysates were treated for 2 h with benzonase nuclease (Merck Millipore) and concentrated with 0.5 M NaCl and 10% weight/volume Polyethylene glycol 8000 during an overnight incubation at 4°C. After centrifugation, the resulting pellet was resuspended in 10 mM Tris pH = 7.5 and treated with equal volumes of chloroform. DNase and RNase were added, and the mixture was incubated at 37°C for an hour. Proteinase K and 10% SDS were then added for 20 min at 56°C. DNA was extracted with equal volumes of phenol/chloroform/isoamyl alcohol (25:25:1) followed by chloroform/isoamyl alcohol (24:1) extraction. Finally, DNA precipitation was achieved by adding 0.1 volume of 3 M sodium acetate and 2.5 volumes of 100% ethanol, followed by incubation at −20°C for 1 h. Samples were then centrifuged 30 min at top speed and 4°C and the resulting pellet washed with ice cold 75% ethanol, air dried, resuspended in 50 μl of 10 mM Tris pH = 8 and stored at −20°C.

Bacterial DNA was extracted using the Wizard Genomic DNA purification kit (Promega) according to the manufacturer’s recommendations.

### Libraries preparation and sequencing

In order to obtain 500 bp fragments, five cycles (21 s ON, 90 s OFF) of sonication with Bioruptor Pico (Diagenode) were performed on the DNA samples. The libraries were then prepared using the NEBNext Ultra II DNA Library Prep (NEB) kit, in accordance with the manufacturer’s recommendations. Indexing was performed using the NEBNext Multiplex Oligos for Illumina kit (Dual Index Primers Set 1) (NEB) according to the manufacturer’s instructions. Shotgun sequencing (2×150 bp) was carried on a NextSeq 2000 System (Illumina).

### Bacteria and phage annotation

The genome of *E. coli* MEc1 (RefSeq GCF_900199625.1) was re-annotated using Bakta version 1.9.1 using default parameters [32].

The genome of phage LF82_P10 (GenBank LT717094.1) was re-annotated using pharokka version 1.7.1 with prodigal-gv as a gene caller (-g prodigal-gv) [33, 34].

### Mutation analysis

Raw sequencing reads were first cleaned using fastp version 0.23.4 [35] with the following parameters: *--trim_tail1 3 --trim_tail2 3 --trim_poly_g --trim_poly_x -r -W 4 -M 20 -u 30 -e 20 -l 100*. The cleaned reads were then processed using Breseq version 0.38.1 [36], aligned to their respective reference genomes, either downloaded from NCBI or re-annotated as described earlier. Then, Breseq output was processed with multiple filters to facilitate downstream analysis. First, clones lacking sufficient coverage in Breseq were excluded from further analysis. Clones 13 and 14 were also excluded due to Breseq errors indicating potential contamination by other bacteria. Next, mutations common to all clones within a given bacterial or phage strain were removed, as they likely represent ancestral mutations in the laboratory strains. To focus on mutations most likely to induce phenotypic changes, synonymous mutations and those located in intergenic regions were filtered out from the analysis.

The selection of these mutations was integrated with the results of infectivity tests of each bacteria/phage clone to identify those responsible for host range shifts. Four clones which had a very high number of mutations (>30) were excluded from the analysis. Most clones carried less than 10 mutations. The resulting binary data were used to create binomial generalized linear mixed effects models. Bacteria and phage mutations were modelled separately, and different models testing either the effect of H2O2, or of each bacterial strain, were built to test their effects. The analysis was performed with R version 4.3.2 [37]. The code corresponding to this analysis can be accessed at https://doi.org/10.5281/zenodo.17640478.

## Results

### Oxidative stress decreases the phage-to-bacteria ratio

We studied a community composed of three *E. coli* strains (MG1655, LF82, and MEc1) and the virulent phage LF82_P10 (P10). This phage was originally isolated on *E. coli* strain LF82, but can also infect strain MEc1, albeit with 100-fold lower efficiency of plating (EOP = 10^−2^). However, phage P10 is unable to adsorb to strain MG1655 and hence cannot infect it [24].

**Figure 1:**
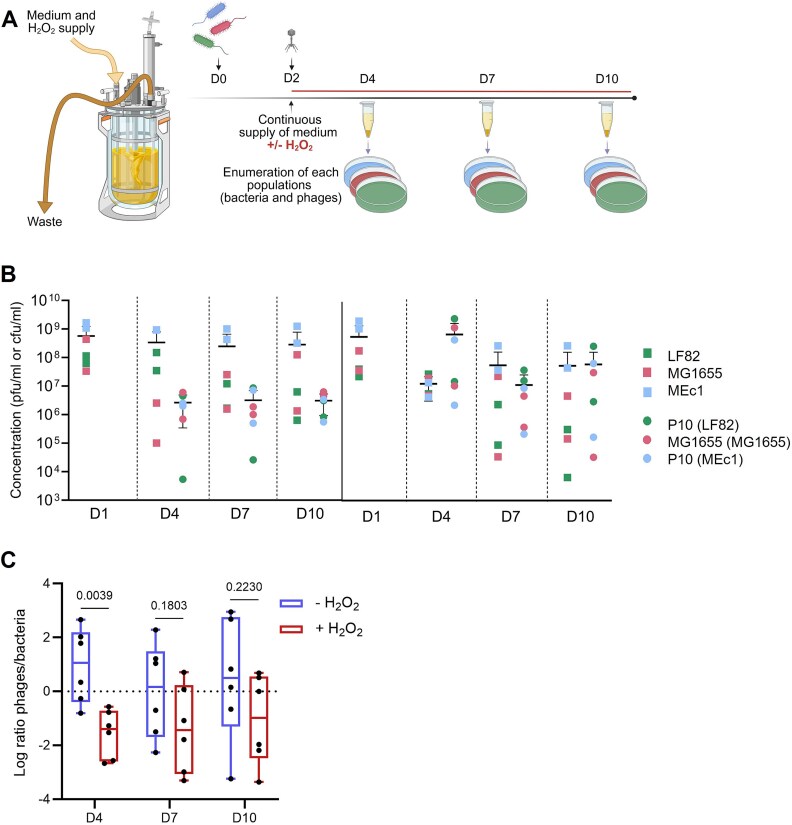
Oxidative stress has no impact on the stability of the model and reduces the relative abundance of virulent phages. (A) Schematic experimental timeline and enumeration of all the populations present within bioreactors at Days 1, 4, 7, and 10 in the absence (*n* = 2) or presence of hydrogen peroxide (*n* = 2). (B) Concentration of each bacterial strain (cfu/ml; squares) and each subpopulation of phages isolated on the three bacterial strains (in brackets) (pfu/ml; circles) at Days 1, 4, 7, and 10 in the absence (− H_2_O_2_) or in the presence (+ H_2_O_2_) of hydrogen peroxide. (C) Comparison of the phage-to-bacteria ratio at Days 4, 7, and 10 in the presence or absence of hydrogen peroxide.

To examine the dynamics of bacterial and phage populations, we monitored their densities over a period of 10 days in two chemostats, each with a continuous supply of medium to enable permanent bacterial and phage replication (Fig. 1A). Bacterial populations were quantified, and phage titres were determined by infection assays on each of the three ancestral *E. coli* strains. We assessed the impact of oxidative stress in two more chemostats by exposing this community to hydrogen peroxide (H_2_O_2_), a ROS playing a pivotal role in the inflammatory response [2] (Fig. 1A). We selected a dose of 500 μM which does not influence phage P10 infectivity (Supplementary Fig. S1A) and has minimal impact on the individual bacterial growth rate, with that of strain LF82 being affected (*P =* .003, Welsh t-test) but not that of strains MG1655 and MEc1 (*P =* .116 and *P =* .066, respectively) (Supplementary Fig. S1B), but without impact on their persistence together in chemostats over time, possibly as the results of cumulative detoxification kinetics. This dose of H_2_O_2_ strongly upregulates the expression of *katG* (catalase-peroxidase) and *dps* (DNA-binding protein), two genes of the OxyR regulon, for all three *E. coli* strains (Supplementary Fig. S1C). Given that KatG is a bifunctional hydroperoxidase I, with both catalase and peroxidase activity, we anticipated a shorter half-life for hydrogen peroxide in chemostats containing bacteria. We measured a degradation time of 15 min for 500 μM H₂O₂ and adjusted the chemostat flow rate accordingly to maintain a constant H₂O₂ concentration in the culture medium and ensuring persistent upregulation of genes *katG* and *dps* in the bacterial community (Supplementary Fig. S1D).

Strain MEc1 was consistently the most abundant population within chemostats, and the abundance of the three *E. coli* strains remained relatively stable over time in both conditions (Fig. 1B and C).

We tested whether phage infectivity towards the three *E. coli* strains had evolved over the course of the experiment, potentially due to phage adaptation to specific hosts or the emergence of resistant bacterial populations. We confirmed that phage P10 adapted to infect strain MG1655, which is consistent with our previous observations *in vivo*, in the murine gut [24], and showed that the same host-range expansion is also reproduced in our *in vitro* model in the presence of oxidative stress (Fig. 1B and C).

The average concentration of total bacteria remained stable in both conditions (no significant difference with a two-way analysis of variance, ANOVA), but the absolute abundance of virulent phages decreased in the presence of hydrogen peroxide, resulting in a decrease in the phage-to-bacteria ratio (Fig. 1C). Because hydrogen peroxide did not affect, per se, phage replication (Supplementary Fig. S1A), we assumed that oxidative stress altered the availability of susceptible sub-populations of bacterial hosts, thereby impacting the phage host range.

### Expansion of phage population diversity is constrained under oxidative stress through phage specialisation

To understand the impact of oxidative stress on phage host range, 80 phage clones were isolated on each bacterial strain (LF82, MEc1, and MG1655) at each of the three time points (Days 4, 7, and 10) from the four chemostats, for a total of 2880 P10 clones. We tested the infectivity of these clones against each of the three ancestral *E. coli* strains. Sub-populations of phages were highly infectious towards strains LF82 and MEc1. Their average infectivity was higher than 85% without hydrogen peroxide and decreased to 71% and 72% in the presence of stress. There was no significant difference in infectivity among different phage sub-populations except for clones isolated on strain MEc1 that showed a higher specialization towards their isolation strain than on strain LF82 in the presence of H_2_O_2_ (Tukey multiple comparison test: *P =* .0249) (Supplementary Fig. S2A and B). By contrast, clones isolated on the new host strain MG1655 showed high specialization towards their isolation strain under both conditions. Indeed, we observed a significant decrease in mean infectivity between clones isolated on this strain and those isolated on strains LF82 and MEc1 (Tukey multiple comparison test: *P =* .0022 and *P =* .0009 without H_2_O_2_ and *P =* .0013 and *P =* .0019 with H_2_O_2_, respectively), resulting in an average infectivity of around 40% (Supplementary Fig. S2C).

**Figure 2:**
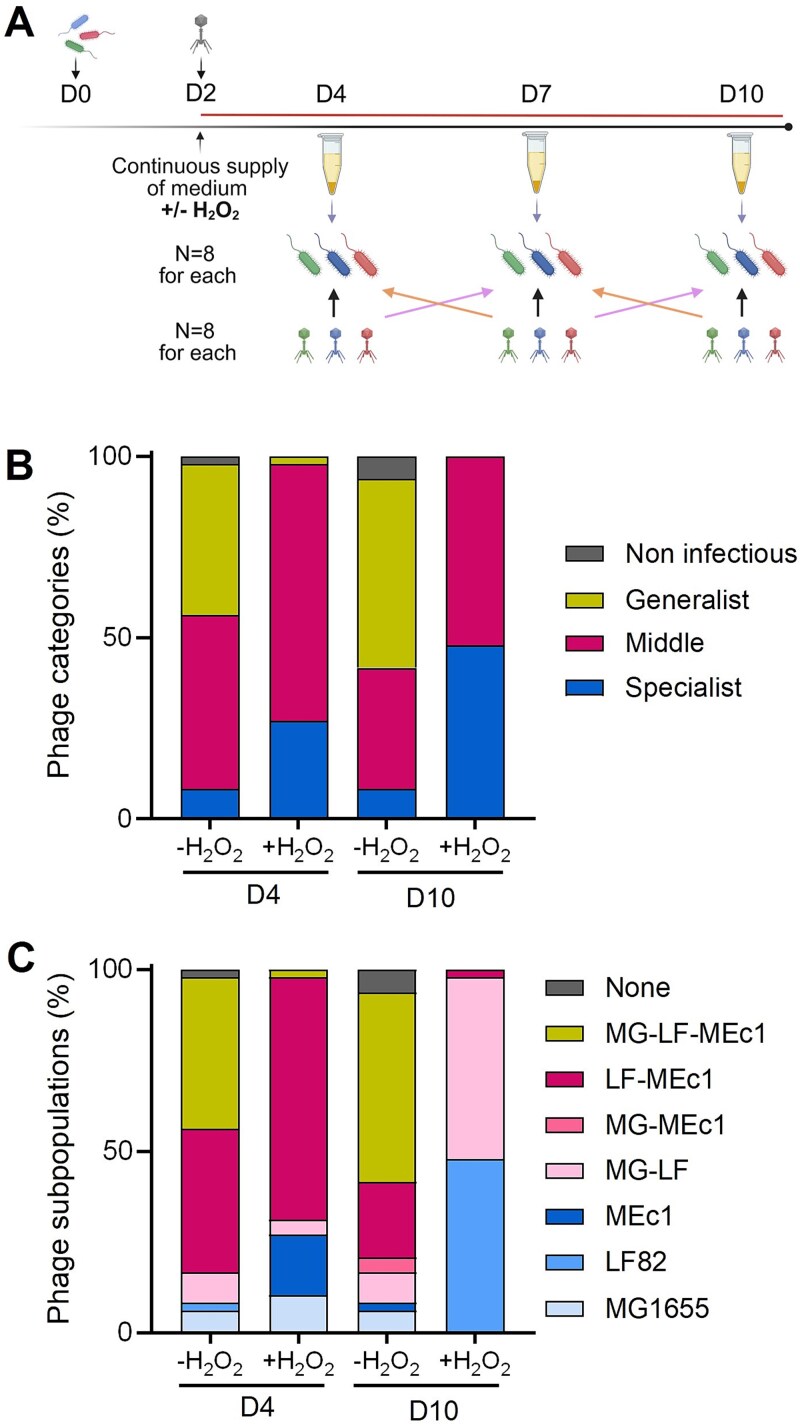
Oxidative stress induces P10 specialisation by reducing subpopulation diversity and host range. (A) Schematic representation of the time-shift experiment showing present, past, and future interactions between the eight clones of each bacterial strain and the eight clones of each phage subpopulation at Days 4, 7, and 10 (i.e. 1728 cross-tests). (B) Percentage of the three categories (specialist: one strain infected, middle: two strains infected, generalist: three strains infected) of phage subpopulations in the absence or presence of hydrogen peroxide at Days 4 and 10. (C) Percentage of the phage subpopulations within the three categories according to the bacterial strains infected in the absence or presence of hydrogen peroxide at Days 4 and 10.

We hypothesised that the specialisation observed towards ancestral strains reflected the evolution of narrower host spectra over time in chemostats. To test this hypothesis, we performed a time-shift experiment. We selected eight clones from the three phage sub-populations at Days 4, 7, and 10 for each condition and each chemostat and tested their infectivity against eight bacterial clones isolated at past, present, and future time points in presence or absence of hydrogen peroxide (Fig. 2A).

Based on time-shift results, we analysed phage infectivity against bacterial strains isolated at contemporary (present) time-points (Fig. 2A; Supplementary Table S1). We classified the host spectrum of each phage clone into three categories representing the number of hosts: specialists (infecting a single bacterial strain), intermediates (infecting two strains), and generalists (infecting all three strains). In control conditions, the three types of spectra are present from Day 4 and maintained stable proportions over time (Fig. 2B). In contrast, under oxidative stress, we observed an increase of the specialist phage populations at the expenses of the generalists. When we considered the diversity of bacterial strains infected, we observed that in the absence of oxidative stress, the presence of diverse bacterial hosts promoted phage diversity, resulting in seven distinct sub-populations dominated by generalists (>50%) with a mix of specialists and intermediates (Fig. 2C). However, the presence of hydrogen peroxide imposed a different evolutionary path: the number of different sub-populations of P10 reduced from five to three over time, and generalist phages went extinct (Fig. 2C) even though their sensitivity to H_2_O_2_ did not differ from that of specialist ones (data not shown). Furthermore, we looked at which strains were preferentially infected and found that oxidative stress significantly impacted the ability of P10 to infect strain MEc1. Indeed, concerning contemporary interactions at the end of the experiment (D10), more than 90% of P10 clones could only infect the strain LF82 or both strains LF82 and MG1655, but not strain MEc1. Phage P10 had lost its ability to infect this strain due to H_2_O_2_-related impact on strain MEc1 which possibly contributes to higher abundance of this strain in the community.

### Oxidative stress accelerates the evolution of bacterial resistance to phage

Under antagonistic co-evolution, phages are expected to be more infectious towards bacteria isolated at previous time points than those from later time-points, as a result of increasing bacterial resistance and phage adaptation in the population [13, 14]. We used the time-shift approach to verify if these dynamics were taking place in our community (Supplementary Table S1).

**Figure 3:**
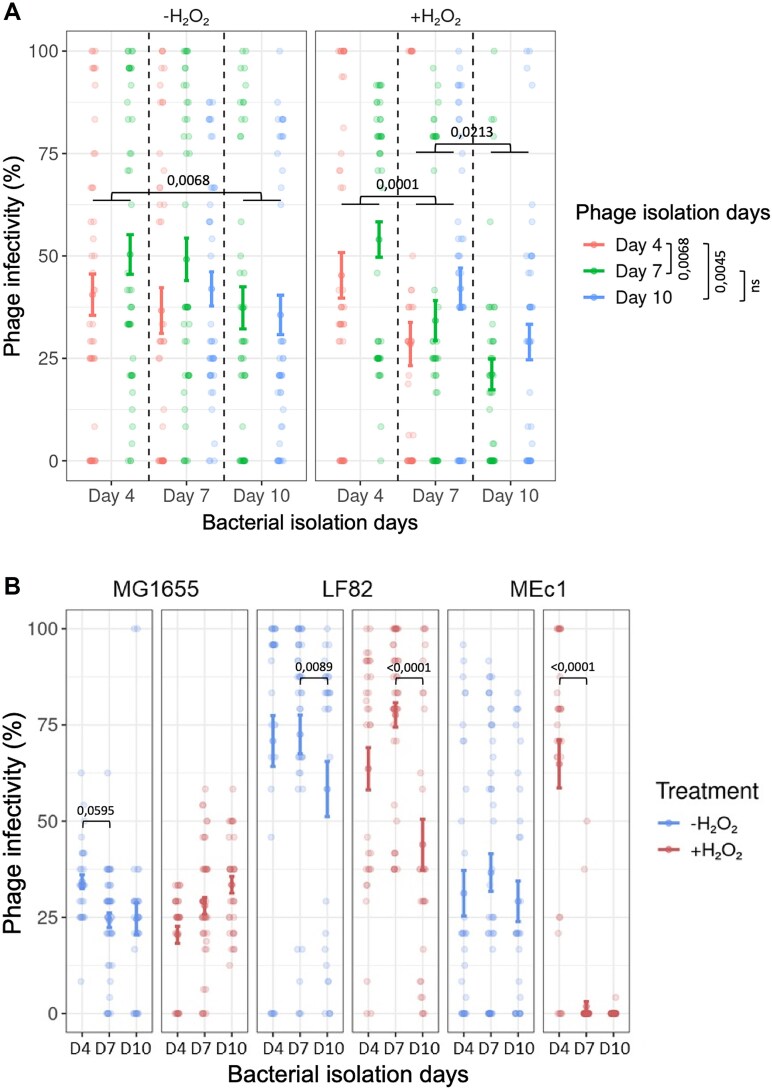
Oxidative stress decreases general infectivity of P10 and boosts evolution, especially for the MEc1 strain. (A) Comparison of the phage infectivity against all the bacteria combined between the two treatments (without and with hydrogen peroxide). The statistical analysis represented the evolution between the different bacterial and phage time points. (B) Comparison of both treatments (without and with hydrogen peroxide) on the evolution of phage P10 infectivity against individual clones of LF82, MG1655, and MEc1 strains isolated at Days 4, 7, and 10.

We compared phage infectivity against the three bacterial strains combined (Fig. 3A) and we focused on the impact of hydrogen peroxide on phage evolution over time. We observed that phage infectivity significantly increased between P10 clones isolated at Days 4 and 7, and between those isolated at Days 4 and 10 (Turkey honestly significant difference test (HSD) HSD: *P =* .0045 and *P =* .0068 respectively), consistent with antagonistic coevolution dynamics. This process then slowed down between Days 7 and 10 (Turkey HSD: *P =* .8778) showing that phage evolution occurs mainly at the beginning of the experiment. Although we did not observe any effect of H_2_O_2_ treatment on the evolution of phage infectivity, bacterial evolution is significantly affected by oxidative stress (ANOVA comparing models with or without bacterial time points and treatment interaction: *P =* .2001 and *P =* .0479, respectively). Indeed, we observed a significant decrease of phage infectivity between bacterial clones isolated at Days 4 and 7, and between Days 7 and 10 in the presence of hydrogen peroxide (Turkey HSD: *P =* .0001 and *P =* .0213 respectively), whereas this decrease was only significant between bacterial clones isolated at Days 4 and 10 in absence of hydrogen peroxide (Turkey HSD: *P =* .0068) (Fig. 3A). These results show that oxidative stress accelerates bacterial evolution, leading to a faster reduction in susceptibility to phage infection under oxidative conditions.

We examined the responses to phage infection for each bacterial strain with and without hydrogen peroxide. We looked at the infectivity of the evolved phages towards the individual bacterial host strains isolated at different time-points. The capacity of P10 to adapt and infect strain MG1655 remained unaffected by oxidative stress throughout the experiment (Fig. 3B; Supplementary Fig. S2B). However, phage infectivity towards strain LF82 and MEc1 was significantly decreased under oxidative stress between Days 7 and 10 compared to the control condition (Tukey HSD: *P* < .0001 and *P =* .0089, respectively) (Fig. 3B). Furthermore, we observed a drastic decrease in the sensitivity of MEc1 to phage infection between Days 4 and 7 (Tukey HSD: *P* < .0001), the whole population becoming nearly completely resistant at Day 10 (Fig. 3B).

Directional temporal shifts in phage infectivity are consistent with the emergence of arms-race mechanisms in both conditions, but oxidative stress accelerates the evolution of bacterial resistance and shapes phage infectivity in a host-dependent way.

### Genomic mutations are associated with the evolution of P10 host range

When antagonistic coevolution between bacteriophages and bacteria follows arms race dynamic, it leads to the selective fixation of genomic mutations conferring bacterial resistance and phage counter resistance. We previously showed that mutations in the *gp90* gene of phage P10, encoding a tail fibre protein, were necessary for adaptation to infect strain MG1655, but bacterial mutations in lipopolysaccharide (LPS) synthesis genes conferred renewed bacterial resistance [25]. Here, we asked how the genomic profiles of both bacteria and phages changed in an evolving community by sequencing four clones from each phage subpopulation and each bacterial strain isolated at the end of the coevolution experiment in chemostats (Day 10).

In bacterial genomes, we found no mutations consistently shared across the three *E. coli* strains, despite them belonging to the same species and sharing a common core genome (Supplementary Table S2), suggesting strain-specific evolutionary pathways or mutation-independent mechanisms involving, for instance, modulation of gene expression. The only mutations shared by two strains were single nucleotide polymorphism (SNP) T43G in the 30S ribosomal gene *rpsL* common to LF82 and MG1655, and mutations in the outer membrane porin OmpC and its regulator OmpR, shared between strains MG1655 and MEc1.

The average number of mutations per bacterial genome was lower in the presence of hydrogen peroxide and we could not correlate specific bacterial mutations with oxidative stress or with resistance to specific phage clones (binomial generalised linear mixed model, see Methods). We asked whether H_2_O_2_ exposure and oxidative stress response pre-selected variants in the populations of the three strains by sequencing amplicons of the most mutated genes after overnight growth, but no mutations in the most targeted genes (*ompR*, *ompC,* and *waaO*) were found (data not shown), suggesting the need for either longer exposure and/or the combined phage pressure.

**Figure 4:**
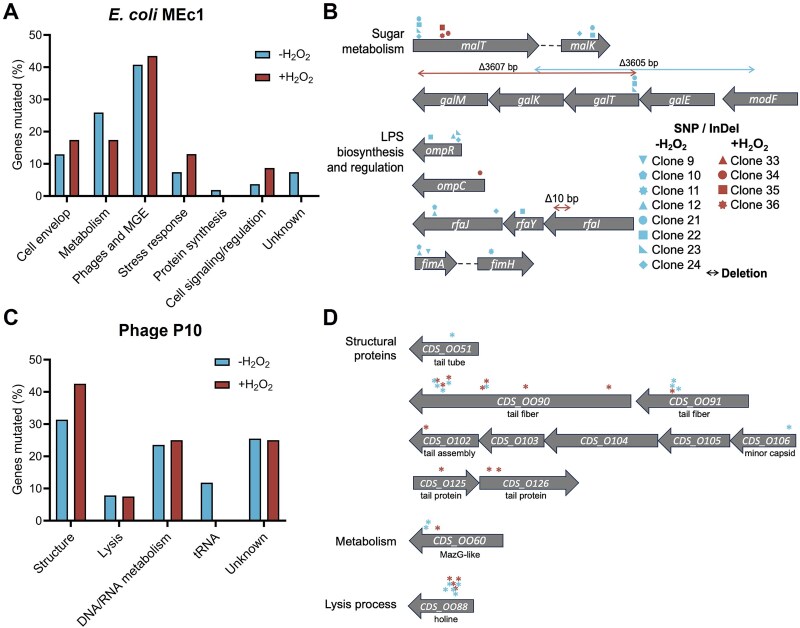
Distribution of the different functions of the mutated genes in absence or presence of hydrogen peroxide in bacteria and phages. Proportional distribution of the functions of the different mutated genes identified at Day 10 in all the sequenced clones of strain MEc1 (A) and phage P10 (C). Mutated genes of interest are represented along with mutations (SNPs, small InDels, and deletions) for strain MEc1 (B) and the phage P10 (D).

We analysed the putative functional impact of the genes carrying non-synonymous mutations to pinpoint functions related to bacterial resistance (Fig. 4 and Supplementary  Fig.  S3). As the MEc1 strain had the most pronounced resistance phenotype, we concentrated on this strain for further analysis.

We observed a large proportion of mutated genes from prophages and other mobile genetic elements (MGEs) (Fig. 4A and B). Although these elements are known to carry defense mechanisms, we could not pinpoint mutations in such functions in MGEs or elsewhere.

Yet, recent findings showed that adsorption is the main determinant of phage host range in *E. coli* [37]. Consistent with this claim, numerous mutations were detected in envelope components that are potential phage receptors. Only clones from strain MEc1 were mutated in genes involved in the biosynthesis of LPS, a receptor for *Felixounaviruses* like phage P10, possibly explaining its higher degree of resistance in the community. MEc1 was also the only strain where we identified specific mutations in the presence or absence of oxidative stress, and all affected the bacterial envelope. In the absence of hydrogen peroxide, targeted genes were involved in the structure of type 1 fimbriae (two SNPs -G24T, C69A- in the gene *fimA* the major subunit and one SNP G87T in *fimH* coding the related adhesin) and LPS biosynthesis (one SNP A220C and a one base insertion in gene *rfaJ* and one SNP T162C in the core heptose kinase gene *rfaY*). Gene *ompR*, regulator of the outer membrane porins OmpC and OmpF, was repeatedly mutated with two SNPs (T90C and A228G) and a two bases deletion, as well as gene *malK*, a maltose ABC transporter ATP binding subunit, part of the maltose regulon and located upstream *lamB,* coding for the receptor of phage lambda.

In the presence of hydrogen peroxide, mutations included SNP G82A in the outer membrane porin OmpC coding gene and a 10 bases deletion in gene *rfaI*, an LPS glucosyltransferase. We also identified several mutations affecting the transcriptional regulator of the maltose regulon MalT in both conditions, suggesting a key role in the coevolving community.

We experimentally tested the functional role of the most abundant mutations. We found that their prevalence in the population is not linked to higher resistance to oxidative stress (Supplementary Fig. S4A). Instead, mutation in LPS biosynthesis conferred resistance to P10 by inhibiting phage adsorption as expected, but this was also true for mutation in the OmpC-MalT genes providing additional insight into the mechanism of P10-host interaction (Supplementary Fig. S4B).

About mutations found in the phage genome, the functional categories of mutated genes were comparable between the two conditions (Fig. 4C and D). However, three structural genes were exclusively mutated in clones isolated under oxidative stress, including SNP G110C in *CDS_102* gene coding for a tail assembly chaperone, SNP C83A in *CDS_125* gene and SNP C65T and a one base deletion at position 85 201 in *CDS_126* gene coding for a tail protein. In absence of hydrogen peroxide we identified several mutations in different tRNAs genes, a SNP G54A in the *CDS_051* gene coding for a tail tube protein and a SNP C57T in the *CDS_106* gene coding for a minor capsid protein. This suggests that most phage mutations were driven by adaptation to varying levels of bacterial resistance in the community, rather than oxidative stress, confirming the results obtained with time-shift experiments. Given that adsorption is the primary limiting step in this system, it was not surprising that most genomic mutations were concentrated in structural genes, particularly those encoding tail fibres, such as *CDS_090*, *CDS_091*, and related genes.

Mutations were also observed in genes potentially associated with counter-defense mechanisms, including *CDS_060* coding for a MazG-like protein, which have been linked to viral counter-defense strategies [38], and tRNA genes [39, 40]. However, the specific role of these genes in P10 infection remains to be demonstrated.

Overall, we show that bacteria exhibit distinct evolutionary responses to oxidative stress, a pattern that is less pronounced in phages. The evolution of phage host range within the microbial community is partially explained by genomic mutations which are likely the results of different infectious dynamics occurring simultaneously on multiple hosts.

## Discussion

Phages and bacteria coexist and co-evolve in various microbial ecosystems, but co-evolution has mostly been studied *in vitro* using simplified phage-bacteria pair models. In natural environments, such as the intestinal microbiota, phage-bacteria interactions are thought to be influenced by the density and diversity of bacteria as well as other phages [18, 23, 41], highlighting the importance of studying these interactions within a community context [41].

We used a previously studied reductionist community [24] and implemented a chemostat model to track the evolution of the host range of a virulent phage in the presence of multiple potential hosts. Chemostats have long been employed to follow medium-to-long term coevolutionary dynamics between phages and bacteria, shedding light on mechanisms of bacterial resistance, phage adaptations, and their associated fitness costs [42–45]. In our study, the community context enabled the expansion of the host spectrum of phage P10 and the emergence of a dominant population of generalist phages. This aligns with previous studies indicating that host range expansion is favoured when novel hosts are highly prevalent in the population and that a broad host range is promoted by high host diversity [46, 47]. However, host range expansion can also involve pleiotropic costs [23, 46, 48]. In our community, beyond the generalist phage population, we identified six additional distinct subpopulations, each differing in abundance and host range, from specialists to generalists. This indicates that in our open system, the presence of multiple hosts not only promoted the evolution of broader phage host ranges but also supported the coexistence of diverse phage populations with varying host specificities.

The parasitic nature of phages means they require a host for isolation, implying that a substantial number of non-infectious particles may remain undetected when using culture-based methods. A strength of this study is that phage sampling was performed across all evolving hosts at various time points, allowing us to capture the maximum diversity. However, a potential limitation is that the sampling may bias the observed proportions of phage populations, as we isolated the same number of clones from bacterial and phage populations present at different densities in the experimental condition. With bacterial and phage densities varying over time we could not account for the different probabilities of phage and host subpopulation encountering which could have influenced the rates of resistance and selection of mutations. When we perturbed the experimental setting by adding hydrogen peroxide, we triggered bacterial stress response and increased resistance to phages, suggesting a general resistant status to multiple stresses. As a result, the phage-to-bacteria ratio was reduced, indicating that inflammation-related ROS indirectly affect the infectivity of virulent phages. This potentially contributes to the expansion of oxygen tolerant species, including pathobionts as *E. coli* and other *Enterobacteriaceae*, and the high temperate-to-virulent phage ratio observed in IBD [4, 5, 49, 50].

A decrease in phage abundance was also linked to reduced host-range diversity, dropping from seven to three infectivity profiles, with many phage populations becoming extinct. Although a decreased bacterial diversity is repeatedly observed in IBD [51, 52], variations in the associated virome are difficult to resolve due to high interpersonal variability [53]. Yet a decrease in viral alpha-diversity was recently shown in a small cohort of Crohn’s disease patients [54]. Our reductionist setting suggests that oxidative stress may be a major contributor in affecting microbiota diversity via modulating phage abundance and host availability.

In our model, resistance to phages developed in a strain-specific way: phages lost the ability to infect strain MEc1, which became hyper-resistance in 10 days of co-culture but persisted in the system by replicating on the susceptible portion of available host strains at lower abundance. Pan-resistance to phage infection in the presence of H_2_O_2_ may stem from a combined unknown mechanisms of stress resistance to both ROS and phages, that would again favour *E. coli* dominance in the microbiota during inflammation.

Consistently, a time-shift experiment showed that coevolution is accelerated in the presence of oxidative stress with a strong effect on phage and bacterial diversity and infection networks.

No clear pattern of mutations was significantly associated with phage infectivity or bacterial susceptibility, likely due to the multiple infection opportunities each phage encounters at any given time depending on the relative abundance and the susceptibility of subpopulations. However, hyper-resistant MEc1 clones—but not the LF82 and MG1655 clones—harboured point mutations in genes involved in various stages of LPS biosynthesis. Similar to other *Felixounaviruses*, phage P10 recognizes an undetermined portion of the LPS as its receptor, and mutations in LPS biosynthesis genes have been previously associated with resistance.

We detected additional mutations that could affect the presence or the structure of outer membrane proteins that classically serve as phage receptors, like OmpC and LamB [11, 43] which were also sufficient to prevent phage P10 adsorption, determining its need for multiple co-receptors. Other mutations may serve as resistance mechanisms against induced temperate *E. coli* phages which were not studied.

The lack of resistance-related mutations in evolved clones from strains LF82 and MG1655 may indicate the existence of other resistance mechanisms, possibly determined by alterations in bacterial physiology affecting phage infectivity as already reported in *E. coli* [19].

We also found a high frequency of mutations in genes carried by prophages and other mobile genetic elements which may also explain the emergence of bacterial resistance within the community. These elements often act as reservoirs for defense mechanisms, enabling bacteria to resist phage infections and acquire immunity [55].

Phage counter-infectivity and adaptation have been linked to specific point mutations in phage P10 structural genes, particularly those encoding tail fibres, which contain receptor-binding domains essential for improving adsorption efficiency.

Genes coding for tRNAs were also often mutated in the control conditions and are known to be associated in fighting bacterial defence mechanisms [39, 40]. Also, a *mazG*-like gene, encoding for a nucleotide pyrophosphohydrolase, was mutated in clones isolated from both conditions. MazG proteins deplete the starvation alarmone (p)ppGpp [38] and affect the toxicity of MazF, as part of the MazE/F toxin-antitoxin system, conserved in *E. coli.* These mutations suggest that intracellular defense mechanisms and abortive infection could also interfere with phage-bacteria interactions in our experimental setup, but their role remains to be demonstrated. In conclusion, we show that oxidative stress strongly affects the interactions between virulent phages and their host bacteria which may play a critical role in the shift in microbiota diversity associated with intestinal inflammation.

## Supplementary Material

Meynard_Doumenc_supplementary_figures_revisions4_wrag090

Meynard_Doumenc_TableS1_wrag090

Meynard_Doumenc_TableS2_wrag090

## Data Availability

The genomic data underlying this article are available in the Sequence Read Archive (SRA, NCBI) under BioProject accession PRJNA1226777.
