# Supplementary material for: Oxidative stress constrains evolution of bacteriophage host-range diversity
Source: ISME J. 2026 Apr 14;20(1):wrag090. doi: 10.1093/ismejo/wrag090 (PMC13155118; doi:10.1093/ismejo/wrag090)
Supplement: Meynard_Doumenc_supplementary_figures_revisions4_wrag090 [file meynard_doumenc_supplementary_figures_revisions4_wrag090.pdf]

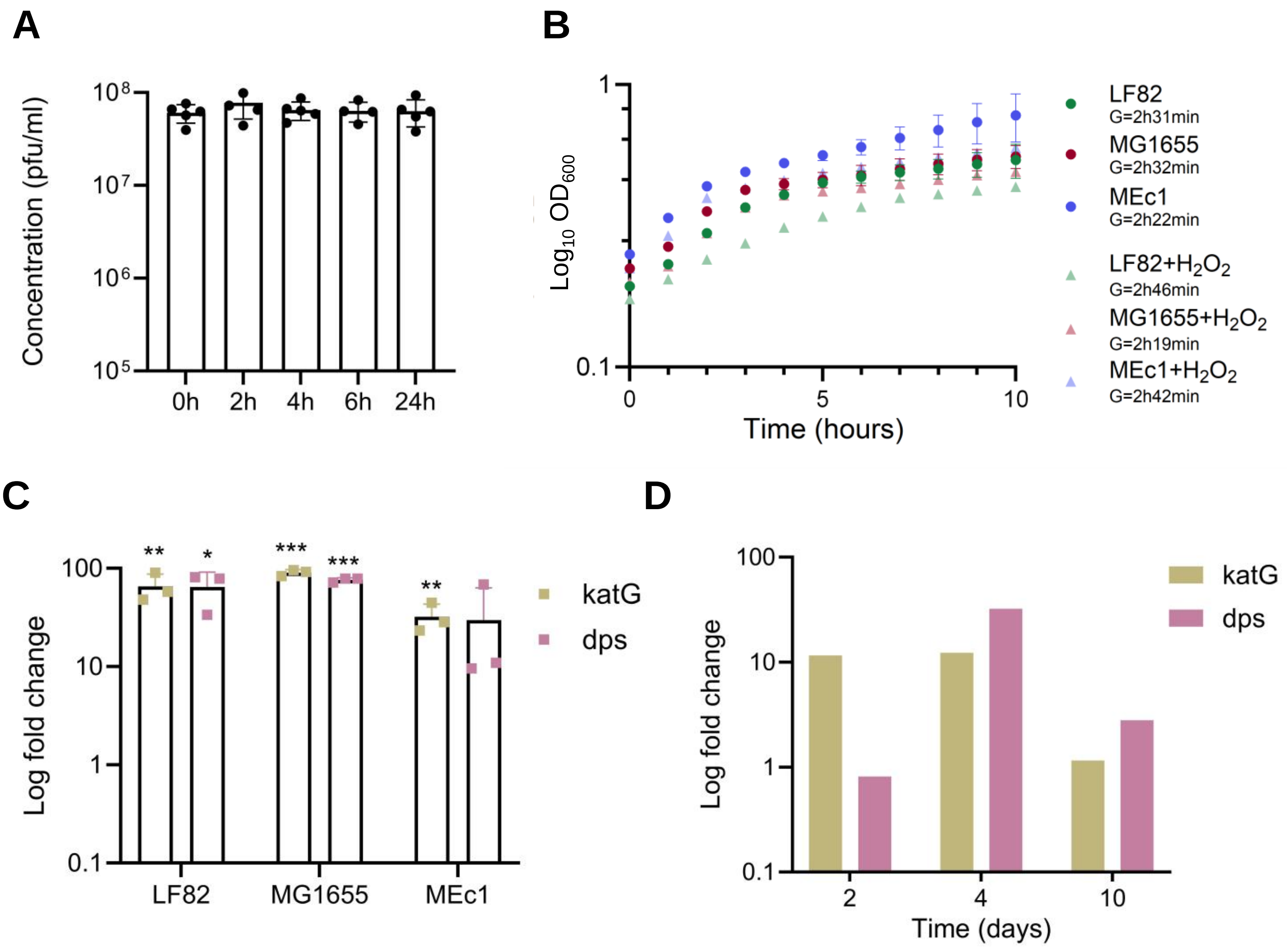

Figure S1: Impact of hydrogen peroxide on the bacterial strains and on P10 infectivity.
A) Titration of LF82_P10 on its host, *E.coli* LF82, before and 2h, 4h, 6h and 24h after addition of hydrogen peroxide.
B) Kinetic of the three *E. coli* strains in absence (round) and in presence (triangle) of 500µM of hydrogen peroxide. Optical density measurement was automatically taken every 15 minutes using a Tecan spectrophotometer after 30 seconds agitation. Data are shown are means of three biological replicate values ± standard error from the mean (s.e.m.) and average generation times (G) are indicated. C and D) mRNA fold change measured by RT-qPCR for katG and dps genes expression (oxidative stress response genes) on the three *E. coli* strains (C) and within bioreactor at day2 (2hours after H2O2 addition) day4 and day10 (D) in presence of hydrogen peroxide compared to basal state. Expression of *katG* and dps normalized with *gapA* (GAPDH) gene expression.
.

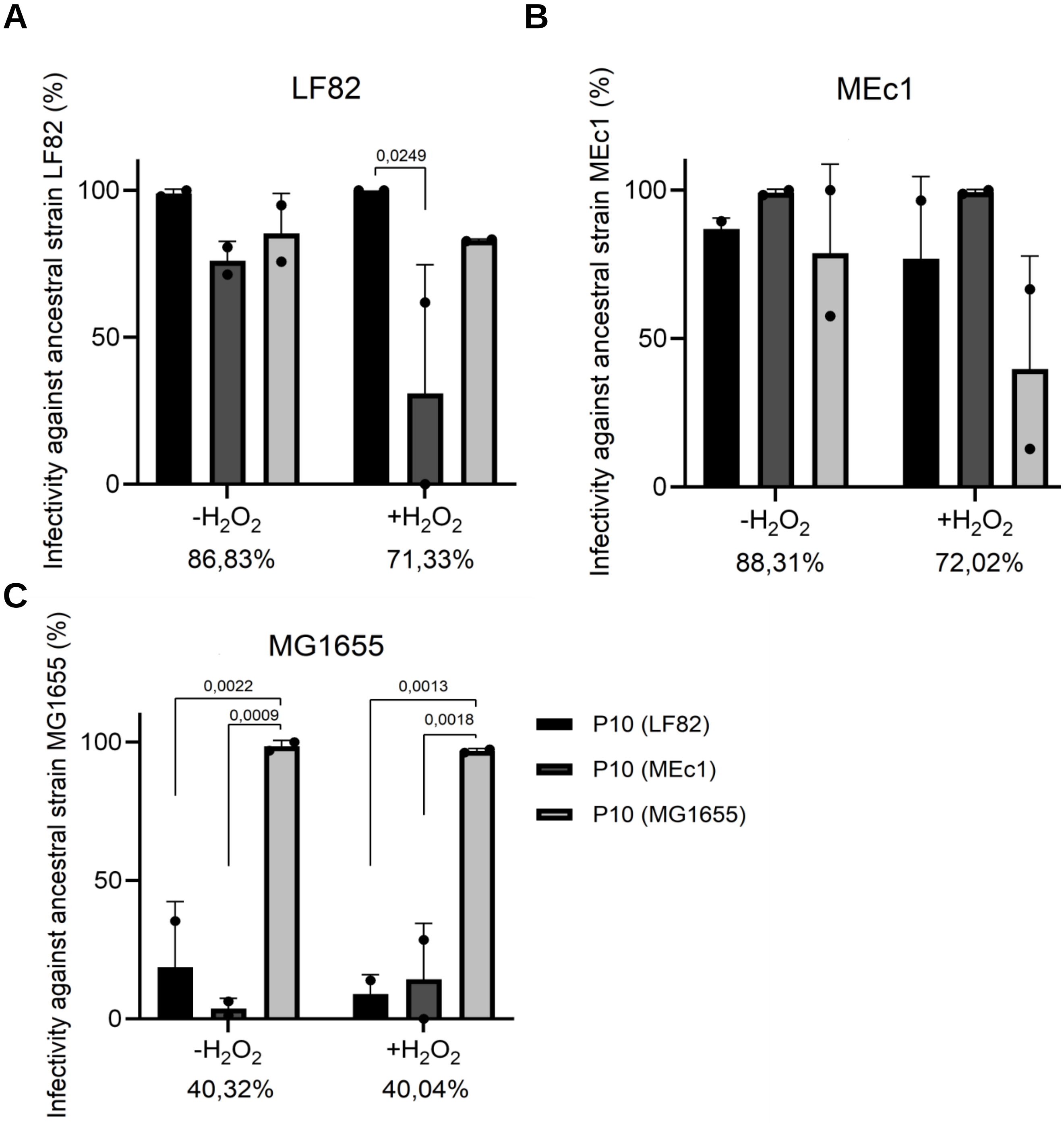

Figure S2: P10 infectivity against ancestral bacterial strains in absence and in presence of hydrogen peroxide. Percentage of infectivity of 80 clones of phage P10 isolated on the three strains of *E. coli* at day 10 tested against the ancestral strains LF82 (A), MEc1 (B) and MG1655 (C) in absence and in presence of hydrogen peroxide. Each point represents the average of the 80 clones tested for each chemostat (n=2). The mean infectivity for the three subpopulations of P10 is shown under each condition (%).

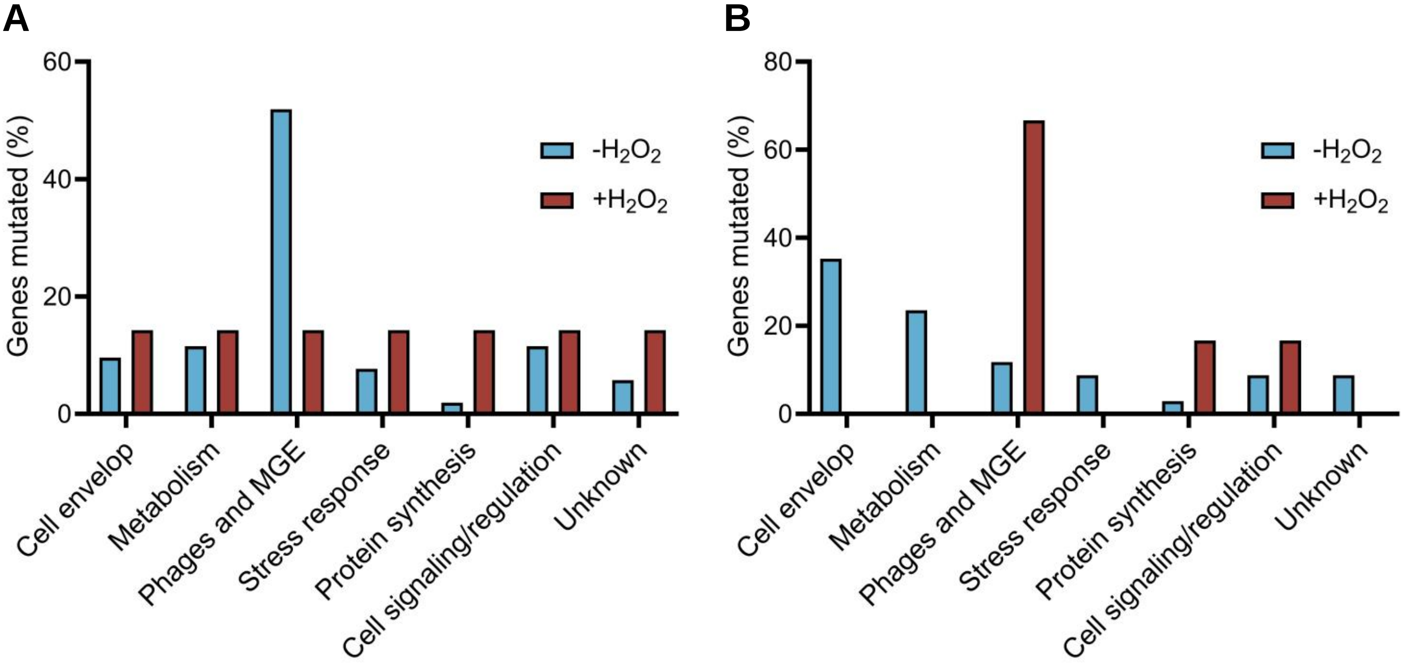

Figure S3: Distribution by bacterial strain of the different functions of the mutated genes identified in absence or presence of hydrogen peroxide. Representation of the proportional distribution of the functions of the different mutated genes identified at day 10 in all the sequenced clones of the strains LF82 (A) and MG1655 (B). For strain LF82, 52 mutated genes were identified in absence and seven in presence of hydrogen peroxide. For strain MG1655, 34 mutated genes were identified in absence and six in presence of hydrogen peroxide.

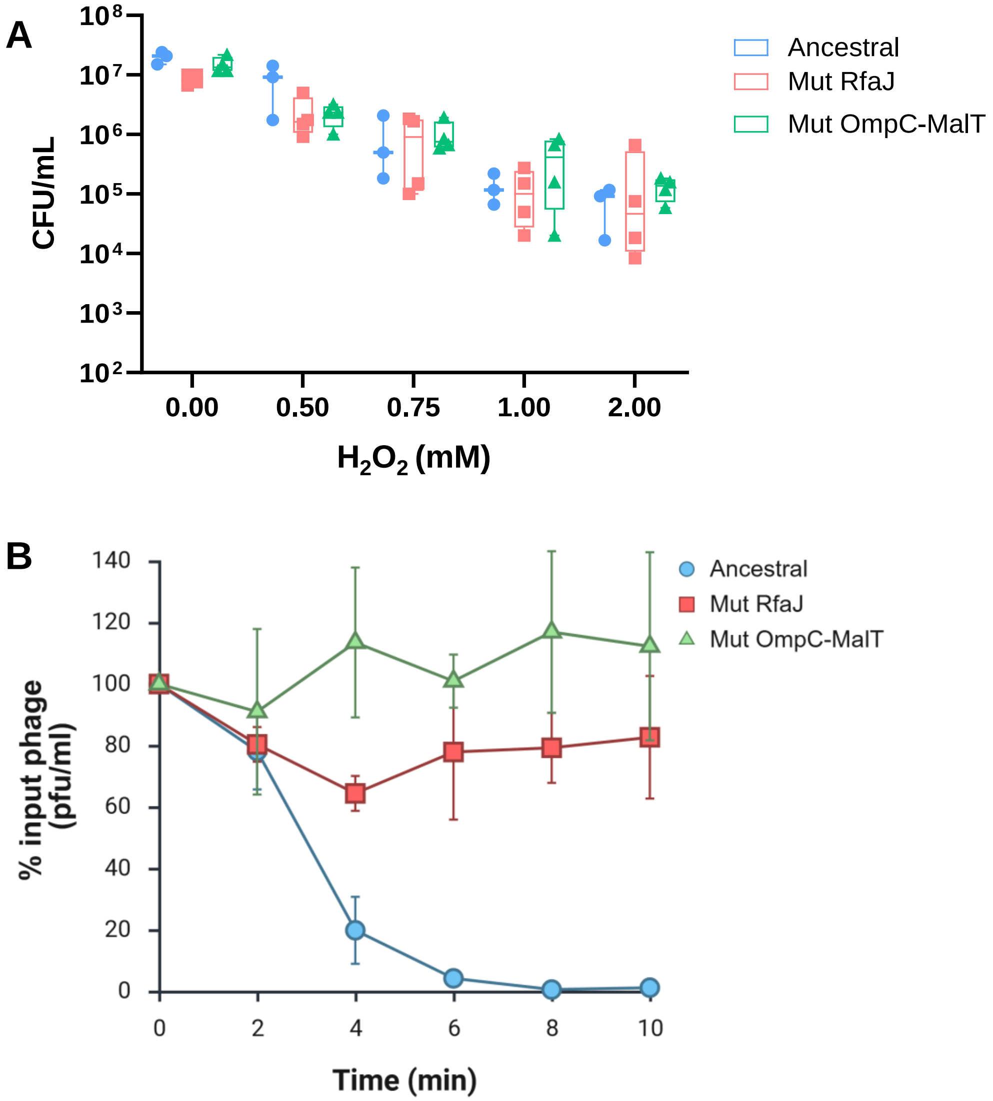

Figure S4: Characterisation of evolved mutant clones of strain MEc1. (A) Sensibility of ancestral strain MEc1 and two phage P10 resistant clones isolated at day 10 mutated in either the *rfaJ* gene (clone 21) or both the *ompC* and *malT* genes (clone 34) to incubation in the presence of increasing doses of H2O2. At each concentration, there is no statistical difference in survival between the ancestral and the mutant clones (Wilcoxon test, n=3 or 4). (B) Adsorption of phage P10 on ancestral strain MEc1 and two phage P10 resistant clones isolated at day 10 mutated in either the *rfaJ* gene (clone 21) or both the *ompC* and *malT* genes (clone 34).
